# Evaluation of sarcoidosis with multiple bone lesions using both bone scintigraphy and FDG-PET/CT: A case report

**DOI:** 10.1016/j.rmcr.2024.102077

**Published:** 2024-06-21

**Authors:** Naohiro Kadoma, Kenichiro Atsumi, Kaoruko Shinbu, Shunichi Nishima, Kakeru Hisakane, Koji Nagata, Masahiro Seike, Takashi Hirose

**Affiliations:** a Department of Pulmonary Medicine and Medical Oncology, Nippon Medical School, Tama Nagayama Hospital, 1-7-1 Nagayama, Tama-shi, Tokyo, 206-8512, Japan; b Department of Pathology, Nippon Medical School Tama Nagayama Hospital, 1-7-1 Nagayama, Tama-shi, Tokyo, 206-8512, Japan; c Department of Pulmonary Medicine and Oncology, Graduate School of Medicine, Nippon Medical School, 1-1-5 Sendagi, Bunkyo-ku, Tokyo, 113-8603, Japan

**Keywords:** Granuloma, Sarcoidosis, Bone diseases, Radionuclide imaging, Positron-emission tomography

## Abstract

Sarcoidosis is a systemic granulomatous disease; however, the incidence of bone sarcoidosis is relatively rare. The short tubular bones of the hands and feet are most frequently affected, while the vertebrae and the pelvic bones are rarely involved. We hereby report a rare case of multiple bone sarcoidosis involving the vertebrae and pelvic bones, evaluated before and after steroid therapy using two different imaging modalities: bone scintigraphy and A 18F-fluorodeoxyglucose positron emission tomography combined with computed tomography (FDG-PET/CT). FDG-PET/CT is effective for detecting bone lesions; however, whole-body imaging is recommended to detect the short tubular bones of the hands and feet, which are most frequently affected.

## 1 Introduction

Sarcoidosis is a systemic granulomatous disease, and the incidence of bone sarcoidosis ranges from 1 % to 13 % [1]. The short tubular bones of the hands and feet are most frequently involved, while the vertebrae and pelvic bones are rarely involved [2]. Bone scintigraphy is useful for detecting undiscovered bone involvement in the whole body, even in patients with asymptomatic bone sarcoidosis. Recently, A 18F-fluorodeoxyglucose positron emission tomography (FDG-PET) has been reported to assess the extent of involvement and quantify disease activity more accurately in sarcoidosis compared with 67Ga scintigraphy [3]. However, a comparison between bone scintigraphy and FDG-PET for bone sarcoidosis has not yet been performed. We hereby report a rare case of multiple bone sarcoidosis involving the vertebrae and pelvic bones, with evaluations performed using two different imaging modalities: bone scintigraphy and FDG-PET combined with computed tomography (FDG-PET/CT).

## 2 Case presentation

**Fig. 1:**
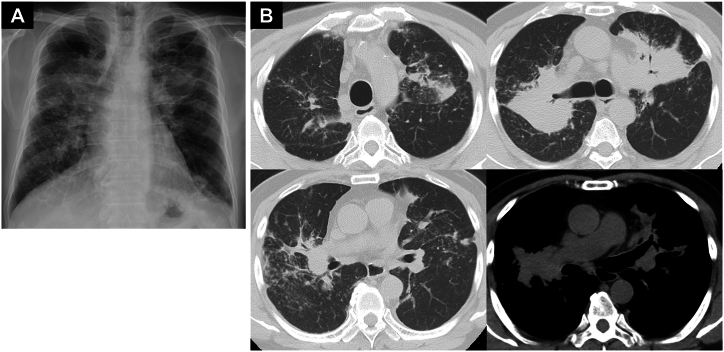
(A) Chest X-ray demonstrated tumor-like lesions in both lungs. (B) Chest computed tomography scan at the first examination demonstrated mediastinal and hilar lymph node swelling, bilateral tumor-like lesions, and granular shadows.

**Fig. 2:**
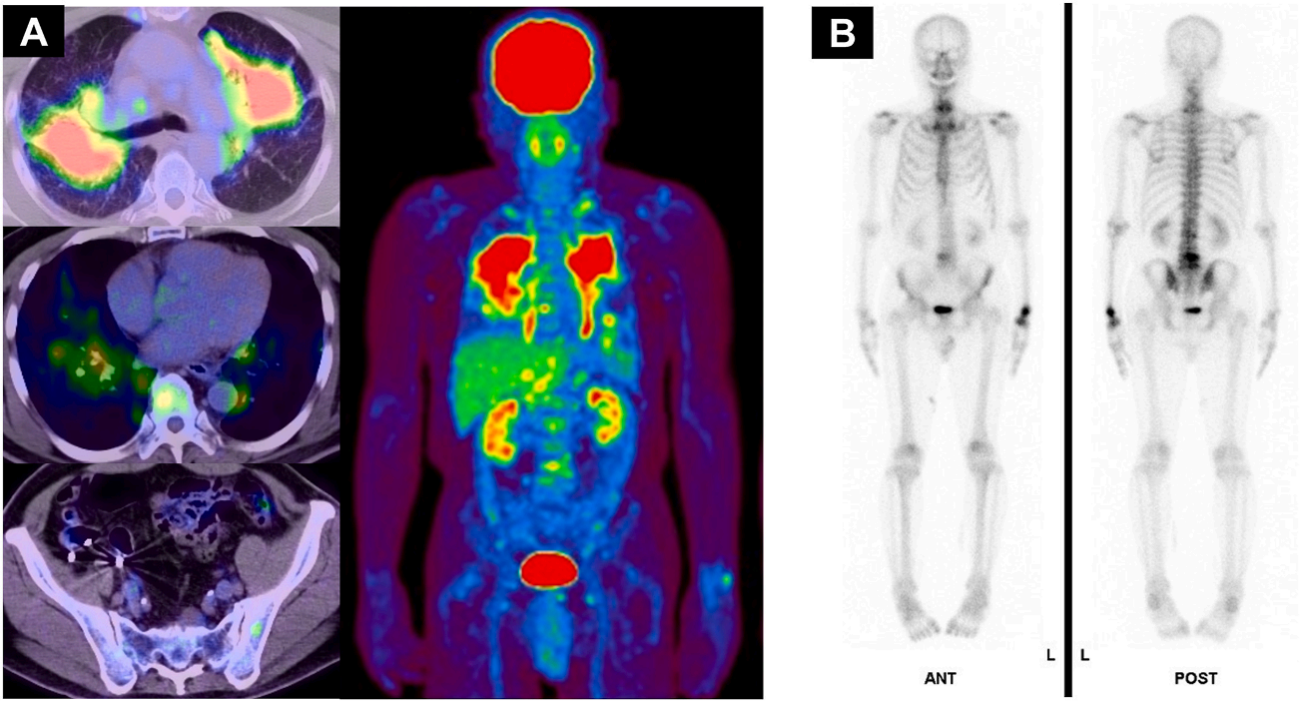
(A) Pretreatment FDG-PET/CT demonstrated multiple areas of uptake in the lungs, lymph nodes (cervical, supraclavicular, mediastinal, hilar, paraaortic, iliac, and inguinal lymph nodes), and multiple bones (thoracic vertebrae, lumbar vertebrae, and pelvic bone). (B) Pretreatment bone scintigraphy demonstrated uptake in the lumbar vertebrae and left carpal bone.

The patient was a 78-year-old Japanese male with no history of smoking who visited our hospital with complaints of increasing dyspnea and bilateral abnormal shadows on a routine chest X-ray (Fig. 1A). He had a history of hypertension and dyslipidemia at the age of 63 years. He worked as a security guard, and he did not have any pets. Blood tests revealed elevated levels of angiotensin-converting enzyme (ACE) (36.6 IU/L; normal range: 7.0–25.0 IU/L) and soluble interleukin-2 receptor (sIL-2R) (1621 U/mL; normal range: 122–496 U/mL) (Table 1). Chest CT revealed mediastinal and hilar lymph node swelling, bilateral tumor-like lesions, and granular shadows (Fig. 1B). FDG-PET/CT revealed multiple areas of uptake in the lungs, multiple lymph nodes (cervical, supraclavicular, mediastinal, hilar, paraaortic, iliac, and inguinal lymph nodes), and multiple bones (thoracic vertebrae, lumbar vertebrae, and pelvic bone) (Fig. 2A). The FDG-PET/CT imaging range did not cover the hands and the feet. Therefore, imaging evaluation with bone scintigraphy using ^99m^Tc-HMDP was also performed. Bone scintigraphy revealed uptake in the lumbar vertebrae and in the left carpal bone, which was not observed in the usual FDG-PET/CT scan (Fig. 2B). Hand X-ray indicated no punch-out lesions. The 12-lead electrocardiogram and echocardiography revealed no abnormalities.

**Table 1:** Laboratory data.

**Hematology**		KL-6	1272 U/mL
WBC	5300/μL	SP-D	110.3 ng/mL
Neutro	58.6 %	HbA1c	6.5 %
Lymph	18.1 %	ACE	36.6 U/L
Eosino	9.3 %	CEA	3.2 ng/mL
Mono	13.0 %	SLX	35.8 ng/mL
Baso	1.0 %	CYFRA	1.6 ng/mL
RBC	486 × 10^4^/μL	Pro-GRP	48.3 pg/mL
Hb	14.6 g/dL	NSE	17.4 ng/mL
Plt	44.4 × 10^4^/μL	sIL-2R	1621 U/mL

**Biochemistry**		**Infection**	
Alb	4.1 g/dL	T-SPOT	(－)
AST	23 U/L	Anti-MAC antibody	<0.5 U/ml
ALT	19 U/L	HIV	(－)
LDH	227 U/L	β-D-glucan	3.9 pg/mL
CK	133 U/L		
BUN	18.7 mg/dL	**Serology**	
Cre	0.84 mg/dL	RF	4 IU/mL
Na	140 mmol/L	* *Antinuclear antibody	<40 mg/dL
K	4.1 mmol/L	MPO-ANCA	0.4 IU/mL
CRP	0.40 mg/dL	* *PR3-ANCA	1.2 IU/mL

**Fig. 3:**
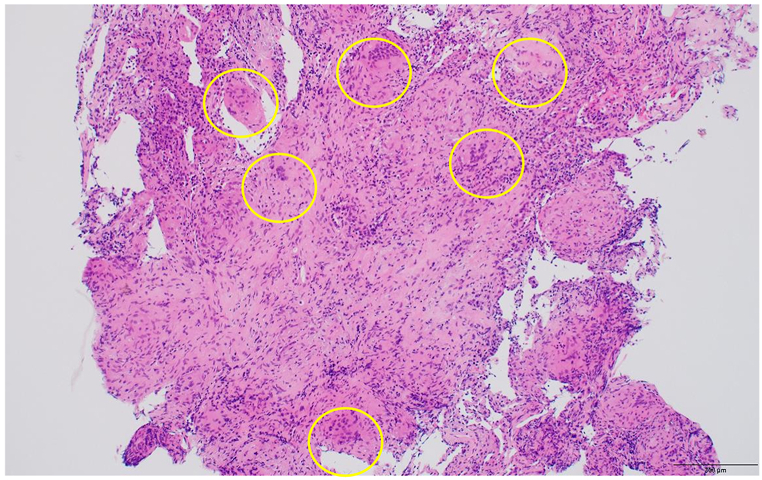
Hematoxylin-and-eosin staining demonstrated noncaseating epithelioid cell granulomas with multinucleated giant cells on lung and fibro connective tissues (area surrounded by yellow frame). (For interpretation of the references to colour in this figure legend, the reader is referred to the Web version of this article.)

A bronchoscopy was performed in the hospital. Bronchoalveolar lavage (BAL) was performed in the inferior segment, and the total recovery rate of BAL fluid was 63.8 % (83/130 ml). The lymphocyte count in BAL fluid was 22 %, with no significant increase, and CD4+/CD8+ lymphocyte ratio was 1.1. Transbronchial lung biopsy performed on the left upper lobe revealed noncaseating epithelioid cell granulomas with multinucleated giant cells on lung tissues and fibro connective tissues (Fig. 3).

**Fig. 4:**
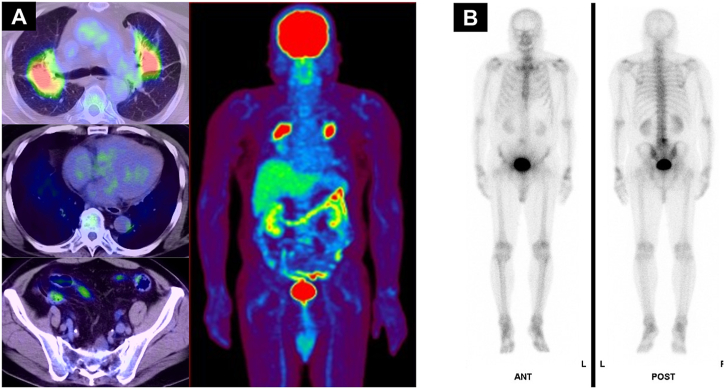
(A) After treatment, FDG-PET/CT indicated that the shadows in the lung field had decreased, and the uptake of bone lesions had almost disappeared. (B) After treatment, bone scintigraphy demonstrated decreased uptake of bone lesions.

A diagnosis of sarcoidosis with multiple bone lesions was made based on radiological, histopathological, and blood test findings, and the findings of ophthalmological examination, which indicated uveitis and retinal perivasculitis. Since the patient had respiratory discomfort, he was initiated on a once-daily oral treatment with 30 mg of prednisone. The dose of prednisone was tapered by 5–10 mg, with continuous monitoring of chest X-ray imaging and serum ACE levels. The symptoms of respiratory disturbance showed marked improvement following six months of steroid therapy. Follow-up FDG-PET/CT indicated that the shadows in the lung field had decreased, and the uptake of bone lesions had almost disappeared (Fig. 4A). Bone scintigraphy also demonstrated decreased uptake of bone lesions (Fig. 4B). Currently, the patient is receiving steroid therapy with 5 mg of prednisone.

## 3 Discussion

We have reported a rare case of multiple bone sarcoidosis involving the vertebrae and pelvic bones. To the best of our knowledge, this is the first report describing a case of multiple bone lesions with evaluations performed before and after steroid therapy using two imaging techniques, bone scintigraphy and FDG-PET/CT.

The short tubular bones of the hands and feet are most frequently involved in bone lesions of sarcoidosis and the vertebrae or pelvic bones are rarely involved. Table 2 summarizes previously reported cases of sarcoidosis with vertebral bone lesions, including the present case [[4], [5], [6], [7], [8], [9], [10], [11], [12], [13], [14], [15], [16], [17], [18]]. Among the 16 cases reported, 11 were symptomatic, and the symptom observed in all these cases was pain. The American thoracic society/European respiratory society guidelines do not indicate the best treatment choice for bone sarcoidosis [19]. Bone lesions are reportedly more prone to fractures, but asymptomatic lesions usually do not need to be treated. It is not known when steroid therapy should begin or whether only symptomatic cases should be treated. Steroid therapy can lead to osteopenia and hence careful follow-up combined with preventive treatment for osteoporosis is strongly recommended. In the present case, steroid therapy was required because of respiratory disturbance associated with the lung lesions. Among the 16 cases reported, corticosteroids were administered in 11 cases, and the pain associated with bone lesions improved in most cases. Therefore, these results support the administration of steroid therapy to prevent disability in cases of bone sarcoidosis with severe symptoms.

**Table 2:** Clinical characteristics of patients with sarcoidosis having vertebral bone lesions reported in previous studies and including our patient.

No	Author	Year	Age/Sex	Other bone lesions	Other organ lesions	Modality of bone imaging	Pain	Steroid therapy
1	Nanda [4]	2007	44/F	–	Lung, lymph nodes	MRI	–	–
2	Morgan [5]	2008	48/M	–	Lymph nodes, spleen	MRI	+	+
3	Hameed [6]	2011	41/F	–	Lymph nodes	MRI	+	–
4	Mehrotra [7]	2011	59/F	rib, pelvis	Lung, lymph nodes, liver	MRI, X-ray	+	+
5	Fujimoto [8]	2013	73/F	–	Lung, lymph nodes, liver, spleen	PET/CT, MRI	+	+
6	Acar [9]	2015	55/F	pelvis	Lung, lymph nodes	PET/CT	+	+
7	Makis [10]	2018	60/F	skull, rib, humerus, sternum, pelvis, femur	Lung, lymph nodes, liver, spleen	PET/CT	–	–
8	Bel-Ange [11]	2018	55/F	femur	Lymph nodes	PET/CT, MRI	+	+
9	Kassimi [12]	2020	35/M	pelvis	Lung, lymph nodes	PET/CT, MRI	+	+
10	O'Riordan [13]	2020	43/M	pelvis	Lymph nodes	PET/CT	+	+
11	Sarvesvaran [14]	2021	66/M	skull, femur	Lung, lymph nodes	PET/CT, MRI, bone scintigraphy	–	–
12	Nel [15]	2021	70/F	skull	Lung, Lymph nodes	MRI	–	+
13	Hasbani [16]	2023	54/M	scapula	Lung, lymph nodes	PET/CT	+	–
14	Hamdi [17]	2023	58/F	rib, pelvis	Lung, lymph nodes, liver	MRI	+	+
15	Alelaumi [18]	2023	46/F	rib	Lung, lymph nodes	PET/CT, MRI, X-ray	+	+
16	Present case	2024	78/M	carpus, pelvis	Lung, lymph nodes, eye	PET/CT, bone scintigraphy	+	+

In the present case, both bone scintigraphy and FDG-PET/CT were used to evaluate multiple bone lesions before and after steroid therapy. Traditionally, general organ examination in sarcoidosis has been performed using plain or contrast-enhanced CT and 67Ga scintigraphy. In the recent years, FDG-PET/CT has been proposed to be a useful imaging modality for the diagnosis and management of patients with inflammatory disease, particularly sarcoidosis. A previously conducted review has indicated that whole-body FDG-PET/CT is more sensitive than 67Ga scintigraphy for evaluating the activity of sarcoidosis and can be of great value in detecting occult diagnostic biopsy sites [3]. As 67Ga scintigraphy has a low sensitivity for detecting bone lesions [20], their actual incidence can remain underestimated.

Generally, because of the low incidence of bone lesions in sarcoidosis patients, bone scintigraphy is rarely performed on patients with sarcoidosis. The difference in sensitivity between bone scintigraphy and PET-CT for detecting bone lesions has not been clarified. In this present case, FDG-PET/CT was observed to be slightly better than bone scintigraphy for detecting axial bone lesions. However, whole-body bone scintigraphy was needed to detect the carpal bone lesion, since the carpal bone is not evaluated in routine FDG-PET/CT scans. The imaging ranges of FDG-PET/CT are usually up to the humerus and the base of the femur. The bone lesions most frequently involved in sarcoidosis are the short tubular bones of the hands and feet [2], but these cannot be detected on routine FDG-PET/CT. Therefore, there could be potentially more bone lesions of sarcoidosis. X-ray and MRI are often used for evaluating local lesions of bone sarcoidosis which are associated with symptoms such as pain, stiffness, and difficulty in moving. In the present case, MRI was not used because he did not have any symptoms indicating bone lesions. X-ray examination of the carpal bone did not reveal any bone lesions.

In the present case, bone scintigraphy and FDG-PET/CT conducted before and after steroid therapy were useful in evaluating treatment efficacy. Generally, the uptake of fractures in benign osseous diseases disappears earlier with FDG-PET than with bone scintigraphy. After six months of steroid therapy, the uptake of bone lesions was observed to almost disappear on FDG-PET/CT, but on bone scintigraphy the uptake was found to have been reduced. It was difficult to judge whether PDG-PET/CT reflected the early disappearance of lesion activity or whether bone scintigraphy was more accurate in reflecting slight activity. Therefore, further studies are required in the future to determine the appropriate imaging modality for evaluating treatment efficacy.

Through the present case, we wish to showcase the following two points: First, bone scintigraphy or FDG-PET/CT is recommended to evaluate systemic bone lesions in patients with symptomatic bone lesions. Second, whole-body FDG-PET/CT is particularly recommended as an alternative imaging modality to 67Ga scintigraphy for general organ examination and detection of asymptomatic bone lesions, even in patients with no symptoms.

## 4 Conclusions

We have reported a rare case of multiple bone sarcoidosis involving the vertebrae and pelvic bones, evaluated before and after steroid therapy using both bone scintigraphy and FDG-PET/CT. FDG-PET/CT is particularly effective for detecting bone lesions, but whole-body imaging is recommended to detect the short tubular bones of the hands and feet, which are most frequently affected.

## Funding

This research did not receive any specific grants from funding agencies in the public, commercial, or not-for-profit sectors.

## CRediT authorship contribution statement

**Naohiro Kadoma:** Data curation, Writing – original draft, Writing – review & editing, Formal analysis, Investigation, Project administration. **Kenichiro Atsumi:** Conceptualization, Formal analysis, Writing – original draft, Writing – review & editing, Investigation, Methodology. **Kaoruko Shinbu:** Investigation, Writing – review & editing. **Shunichi Nishima:** Investigation, Writing – review & editing. **Kakeru Hisakane:** Investigation, Writing – review & editing. **Koji Nagata:** Data curation, Visualization, Writing – review & editing. **Masahiro Seike:** Supervision, Writing – review & editing. **Takashi Hirose:** Supervision, Writing – review & editing, Project administration.

## Declaration of competing interest

The authors declare that they have no known competing financial interests or personal relationships that could have appeared to influence the work reported in this paper.
